# FDA Approval Summary: Ribociclib Indicated for Male Patients with Hormone Receptor-Positive, HER2-Negative Advanced or Metastatic Breast Cancer

**DOI:** 10.1158/1078-0432.CCR-23-1133

**Published:** 2023-12-15

**Authors:** Jennifer J. Gao, Christy L. Osgood, Zhou Feng, Erik W. Bloomquist, Shenghui Tang, C. J. George Chang, Tiffany K. Ricks, Sherry C. Hou, William F. Pierce, Donna R. Rivera, Richard Pazdur, Paul G. Kluetz, Laleh Amiri-Kordestani

**Affiliations:** 1 Oncology Center of Excellence, Research, U.S. Food and Drug Administration, Silver Spring, Maryland.; 2 Center for Drug Evaluation Research, U.S. Food and Drug Administration, Silver Spring, Maryland.

## Abstract

On December 10, 2021, the FDA expanded the indications for ribociclib to include male patients for the treatment of hormone receptor (HR)-positive, HER2-negative advanced or metastatic breast cancer. Ribociclib is now indicated in combination with an aromatase inhibitor (AI) as initial endocrine-based therapy in adult patients, or with fulvestrant as initial endocrine-based therapy or following disease progression on endocrine therapy (ET), in postmenopausal women or in men. The efficacy of ribociclib+AI for male patients was primarily based on previous favorable benefit-risk assessments of ribociclib from MONALEESA-2 and MONALEESA-7 trials, and supported by COMPLEEMENT-1, an open-label, single arm, multicenter clinical trial, in which 39 male patients (n=3,246 total patients) received ribociclib+letrozole+goserelin/leuprolide. The ORR based on confirmed responses in male patients with measurable disease at baseline was 46.9% (95% CI: 29.1, 65.3), consistent with an ORR 43.6% (95% CI: 41.5, 45.8) in the overall population. Overall, adverse reactions occurring in male patients were similar to those occurring in female patients treated with ribociclib+ET. The efficacy of ribociclib+fulvestrant for male patients was primarily based on the previous findings of a favorable benefit-risk assessment from the MONALEESA-3 trial, supported by FDA review of clinical data of a limited number of male patients treated in clinical practice receiving ribociclib+fulvestrant. The known mechanism of action, biologic rationale, and clinical information available adequately demonstrate that the efficacy and safety of ribociclib+AI/fulvestrant are similar in male and female patients. This article summarizes the FDA’s decision-making and data supporting the approval of ribociclib in male patients with breast cancer, and discusses regulatory insights.

## Introduction

Male breast cancer is rare, with approximately 2800 new cases and 530 deaths estimated in 2023.^1^ In contrast, there are an estimated 297,790 new cases of and 43,700 deaths from female breast cancer in 2023.^2^ The majority of male patients are diagnosed in their mid-60s, and most tumors (>99%) are hormone receptor (HR)-positive.^3^ Although the incidence of male breast cancer has increased over the past few decades, it remains a rare disease, accounting for less than 1% of all cancers in men and approximately 1% of all breast cancers.^3^

Historically, male patients have been excluded from participation in clinical trials of breast cancer therapies because the incidence and prevalence are low. This has led not only to limited FDA-approved therapies for male patients with breast cancer, but also the reliance on extrapolated data from the treatment of female patients with breast cancer to inform clinical management of male patients with breast cancer. In August 2020, the FDA Oncology Center of Excellence (OCE) released a final guidance document entitled “Male Breast Cancer: Developing Drugs for Treatment”, encouraging the inclusion of both male and female patients in clinical trials of breast cancer.^4^ For certain clinical trials of oncology products where inclusion of male patients has been rare or none at all, it may be possible to extrapolate the findings to include male patients in the FDA-approved indication, if no difference in efficacy or safety is expected based on the mechanism of action, using data from earlier stages of development, literature, or both. In situations where there may be a concern for differential efficacy or safety between male and female patients, additional supportive data may be generated through a variety of trial designs using different data sources.^4^

Ribociclib, a cyclin-dependent kinase (CDK) 4 and 6 inhibitor (CDKI), was first approved on March 13, 2017, in combination with an aromatase inhibitor for the treatment of postmenopausal women with HR-positive, HER2-negative advanced or metastatic breast cancer, based on the MONALEESA-2 study^5^ (Table 1). On July 18, 2018, the indication for ribociclib in combination with an aromatase inhibitor was expanded to include pre- and perimenopausal women, and ribociclib also received approval in combination with fulvestrant for the treatment of postmenopausal women, as initial endocrine based therapy or following disease progression on endocrine therapy, based on the MONALEESA-7 and 3 studies, respectively^5^ (Table 1). In this article, we present the FDA’s rationale for the approval of ribociclib for use in combination with aromatase inhibitors and fulvestrant for the treatment of male patients with breast cancer, and discuss regulatory insights.

## Clinical Trial Design and Results

The data from the following sources were reviewed to support the expansion of the existing ribociclib indications to include male patients (based on available data at the time of FDA’s review).

MONALEESA-2 was a randomized (1:1), double-blind, placebo-controlled, multicenter clinical trial of ribociclib or placebo in combination with letrozole in postmenopausal women with HR-positive, HER2-negative advanced breast cancer who received no prior therapy for advanced disease^5^ (Table 1). The median duration of exposure to ribociclib plus letrozole was 13 months, with 58% of patients exposed for > 12 months. The trial showed a statistically significant and clinically meaningful improvement in progression free survival (PFS) with the addition of ribociclib to letrozole. The median PFS was not reached (95% CI 19.3, NR) in the ribociclib arm compared to 14.7 months (95% CI 13.0, 16.5) in the placebo arm (HR 0.556, 95% CI 0.429, 0.720, p<0.0001). Male patients were not eligible to enroll in MONALEESA-2.^5^

MONALEESA-3 was a randomized (2:1), double-blind, placebo-controlled trial of ribociclib or placebo in combination with fulvestrant in postmenopausal women, or men, with HR-positive, HER2-negative advanced breast cancer who had received no or only one line of prior endocrine treatment for the treatment of advanced breast cancer^5^ (Table 1). While male patients were eligible for MONALEESA-3, none enrolled. The median duration of exposure to ribociclib plus fulvestrant was 15.8 months with 58% of patients exposed for ≥ 12 months. The trial showed a statically significant and clinically meaningful improvement in PFS and overall survival (OS). The median PFS for ribociclib plus fulvestrant was 20.5 months (95% CI 18.5, 23.5) compared to 12.8 months (95% CI 10.9, 16.3) for placebo plus fulvestrant (HR 0.593, 95% CI 0.480, 0.732, p-value <0.0001). The median OS for ribociclib plus fulvestrant was not reached (95% CI 42.5, NR) compared to 40.0 months (95% CI 37.0, NR) in the placebo plus fulvestrant arm (HR 0.724, 95% CI 0.568, 0.924, p=0.00455).^5^

MONALEESA-7 was a randomized (1:1), double-blind, placebo-controlled trial of ribociclib or placebo plus non-steroidal aromatase inhibitor (NSAI) or tamoxifen plus goserelin in pre- or perimenopausal women with HR-positive, HER2-negative advanced breast cancer who received no prior endocrine therapy and no more than one line of chemotherapy for advanced disease (Table 1).^5^ Due to QT prolongation, ribociclib is not indicated in combination with tamoxifen. The median duration of exposure of ribociclib plus NSAI was 15.2 months, with 66% of patients exposed for > 12 months. In patients who received ribociclib plus NSAI plus goserelin, the trial demonstrated a statistically significant and clinically meaningful improvement in PFS and OS. There was a 13.7-month improvement in median PFS in the ribociclib arm compared to the placebo arm (HR 0.569, 95% CI 0.436, 0.743). Median OS (HR 0.699, 95% CI 0.501, 0.976) was not reached (95% CI NR-NR) in the ribociclib arm versus 40.7 months (95% CI 37.4, NR) in the placebo arm. Male patients were not eligible to enroll on MONALEESA-7.^5^

**Figure 1:**
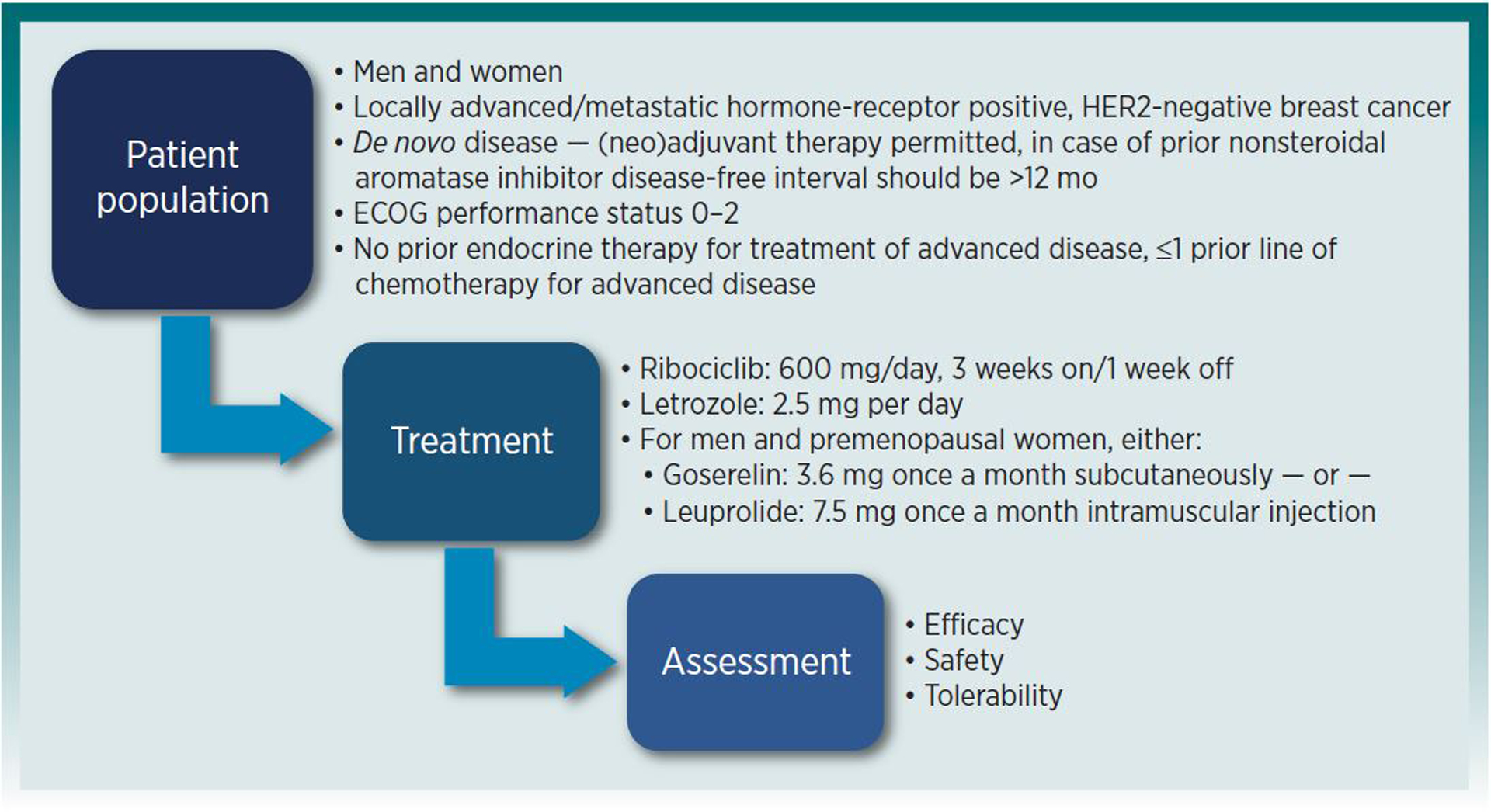
COMPLEEMENT-1 Single Arm Trial Design HER2: human epidermal growth factor receptor 2; ECOG: Eastern Cooperative Oncology Group Source: Created by FDA based on Applicant’s submission

COMPLEEMENT-1 (NCT02941926) was an open-label, multicenter, single-arm trial of ribociclib plus letrozole and goserelin/leuprolide in men and pre- and postmenopausal women with HR-positive HER2-negative advanced breast cancer who had received no prior hormonal therapy but could have received ≤1 line of prior chemotherapy for advanced disease. Patients received ribociclib 600 mg once daily for 21 consecutive days followed by 7 days off plus letrozole 2.5 mg daily continuously, until disease progression or unacceptable toxicity. Goserelin 3.6 mg injectable subcutaneous implant or leuprolide 7.5 mg intramuscular injection were administered on day 1 of each 28-day cycle to men and premenopausal women.^5^ The primary endpoints were safety and tolerability (adverse events, grade 3–4 adverse events, and serious adverse events), and secondary endpoints included time-to-progression (TTP), overall response rate (ORR), clinical benefit rate (CBR), patient reported outcomes, and long-term safety. The trial design is depicted in Figure 1.

COMPLEEMENT-1 enrolled 39 male patients (n=3,246 total patients) between November 30, 2016 and March 22, 2018. The analysis cut-off date was November 8, 2019, and the database was locked on December 19, 2019. As of the cut-off date, 18 (46%) male patients discontinued treatment (11 due to progressive disease, 4 due to adverse events). The median age of male patients was 62 years (range 33 to 80). Of these patients, 39% were 65 years and older, including 10% aged 75 years and older. The male patients enrolled were White (72%), Asian (8%), Black (3%), and 17% unknown. Nearly all male patients (97%) had an ECOG performance status of 0 or 1. Most male patients (97%) had 4 or less metastatic sites, which were primarily bone and visceral (69% each). The median duration of exposure in male patients overall was 20.8 months (0.5–30.6 months, from the start of treatment to last treatment per the cut-off date), with median duration of exposure of 19.2 months to ribociclib (0.5–30.6 months). Twenty-three male patients received ribociclib for 12 months or longer, and 11 male patients received ribociclib for 24 months or longer. The median relative dose intensity was 98.6% for ribociclib (range 87.4–100) and 100% for letrozole. The overall response rate based on confirmed responses in male patients with measurable disease at baseline was 46.9% (95% CI 29.1–65.3, 15/32 male patients, 1 male patient with complete response and 14 male patients with partial response). In confirmed responders with measurable disease at baseline, the median duration of response was not reached. Results are summarized in Table 2. Overall, adverse reactions occurring in male patients were similar to those occurring in female patients treated with ribociclib plus endocrine therapy. The most common adverse reactions (incidence ≥ 20%) were neutropenia, hot flush, diarrhea, arthralgia, fatigue, and asthenia. One male patient died due to grade 3 dyspnea and progression of disease, with documented new lung lesions and a history of tuberculosis. There were four male patients who discontinued treatment due to adverse events (ALT/AST elevation, colon cancer, peripheral edema), and no cases of Hy’s law. There were four cases of grade 1–2 QT prolongation. There was one case of grade 3 QT prolongation requiring dose reduction of ribociclib: this patient ultimately discontinued ribociclib at a later time due to personal reasons. All cases of QT prolongation were identified by ECG monitoring, and QT prolongation is a known adverse reaction of ribociclib, already included in section 5 of the ribociclib labeling as a Warning and Precaution.^5^

## Regulatory Insights

MONALEESA-2, 3, and 7 were large, randomized trials in female patients who received ribociclib/placebo plus hormonal therapy (AI or fulvestrant). Given the rarity of male breast cancer, a randomized trial comparing ribociclib plus hormonal therapy (AI and fulvestrant) vs. placebo plus hormonal therapy would be both impractical and infeasible, as it would take a significant amount of time to enroll and to generate efficacy results. More importantly, it would delay availability of ribociclib, which is not expected to have different efficacy or safety profiles in males compared to females based on the known mechanism of action, to male patients with breast cancer. A significant PFS benefit has already been previously demonstrated in female patients who received ribociclib in addition to hormonal therapy in MONALEESA-2, 3, and 7, with an OS benefit also demonstrated in MONALEESA-3 and 7. Given this demonstrated efficacy benefit of the addition of ribociclib to hormonal therapy in female patients, a trial where male patients are randomized to receive hormonal therapy without ribociclib would lack equipoise.

It is reasonable to rely on efficacy and safety results from the single arm trial of COMPLEEMENT-1 in male patients with breast cancer treated with ribociclib and an AI. Acknowledging the limitations of cross-trial, and cross-patient population, comparisons, ORR in male patients from COMPLEEMENT-1 is comparable with ORR from female patients in MONALEESA-2, 3, and 7, with overlapping 95% confidence intervals across all trials. It is further reasonable to extrapolate findings to expand the indication of ribociclib in combination with fulvestrant to include male patients based on known disease-based biological plausibility, the drug-based mechanism of action of ribociclib, the rarity of male breast cancer, and because no difference in efficacy or safety is anticipated between males and females based on pharmacological mechanism. The Applicant submitted limited contextual real-world data in male patients who received ribociclib in combination with fulvestrant. While no significant safety signals were seen, FDA’s review and approval ultimately relied on the known data and extrapolation from MONALEESA-2, 3, and 7, and COMPLEEMENT-1, given the limited interpretability of clinical information available from the real-world data that was submitted from a very small sample of male patients.

Expanding the existing ribociclib indications in combination with an AI or fulvestrant to include male patients with breast cancer is reasonable, scientifically justified, and consistent with FDA’s guidance on developing therapies for male patients with breast cancer.^4^ Currently, all FDA-approved CDK 4/6 inhibitors, abemaciclib, palbociclib, and ribociclib, are indicated for male and female patients.^5,6^ Male breast cancer is rare and is a serious disease with an ongoing unmet medical need. FDA oncology continues to encourage the thoughtful inclusion of male patients with breast cancer in all clinical trials to increase the evidence available to inform clinical decision making and to potentially support inclusion in a labeling indication.

## Figures and Tables

**Table 1:** Overview of Ribociclib + Hormonal Therapy MONALEESA Clinical Trials (at time of FDA review)

	MONALEESA-2	MONALEESA-3	MONALEESA-7
**Initial FDA regular approval date**	March 13, 2017	July 18, 2018	July 18, 2018
**Hormonal Therapy**	Aromatase inhibitor	Fulvestrant	Aromatase inhibitor
**Eligibility**	Postmenopausal women	Postmenopausal women, Men (none enrolled)	Pre/Perimenopausal women
**CDKI PFS Median** **(months, 95% CI)**	NR (19.3, NR)	20.5 (18.5, 23.5)	27.5 (19.1, NR)
**Placebo PFS Median** **(months, 95% CI)**	14.7 (13.0, 16.5)	12.8 (10.9, 16.3)	13.8 (12.6, 17.4)
**PFS HR** **(95% CI)**	0.556 (0.439, 0.720)	0.593 (0.480, 0.732)	0.569 (0.436, 0.743)
**CDKI OS Median** **(months, 95% CI)**	63.9^1^ (52.4, 71.0)	NR^2^ (42.5, NR)	NR^3^ (NR, NR)
**Placebo OS Median** **(months, 95% CI)**	51.4 (47.2, 59.7)	40.0 (37.0, NR)	40.7 (37.4, NR)
**OS HR** **(95% CI)**	0.765 (0.528, 0.932)	0.724 (0.569, 0.924)	0.699 (0.501, 0.976)

1 Ribociclib USPI updated with MONALEESA-2 OS data on October 3, 2022.

2 Ribociclib USPI updated with MONALEESA-3 OS data on July 6, 2020.

3 Ribociclib USPI updated with MONALEESA-7 OS data on January 21, 2020.

NR = not reached; CDKI = CDK 4/6 inhibitor; PFS = progression free survival; OS = overall survival; HR = hazard ratio; CI = confidence interval

*Source: Ribociclib U.S. Prescribing Information* ^5^

**Table 2:** COMPLEEMENT-1 Summary of Efficacy in Men^1^ (Investigator Assessed, Intent-to-Treat Population)

	Ribociclib + Letrozole + Goserelin or Leuprolide
Overall Response Rate*,^2^	**N = 32**
(95% CI)	46.9 (29.1, 65.3)
**Duration of Response (DoR)** ^3^	**N = 15**
Median (months, 95% CI)	NR (21.3, NR)
Patients with DoR ≥ 12 months, n (%)	12 (80.0%)

Abbreviations: CI, confidence interval, NR, not reached.

* Based on confirmed responses.

1 Patients with measurable disease.

2 Investigator Assessment.

3 Patients with complete response or partial response.

*Source: Ribociclib U.S. Prescribing Information* ^5^
